# Integrated genomics reveals cellular senescence‐driven molecular networks and immune crosstalk in myopia pathogenesis

**DOI:** 10.1002/ccs3.70045

**Published:** 2025-09-21

**Authors:** Wangming Su, Pinsheng Qiu, Yanling Li, Ping Xie, Xiaoyong Yuan

**Affiliations:** ^1^ Ophthalmology Department The Second Hospital of Longyan City Longyan Fujian China

**Keywords:** cellular senescence, gene regulatory networks, immune infiltration, myopia, Tp53‐Cdkn1a‐Myc axis

## Abstract

Myopia, a leading global health challenge linked to severe ocular complications, remains poorly understood in terms of molecular mechanisms involving cellular senescence. This study integrates transcriptomic datasets (GSE112155 and GSE151631) from myopia and normal vision samples to unravel senescence‐driven pathways and immune interactions underlying myopia pathogenesis. By constructing protein–protein interaction networks and post‐transcriptional regulatory axes (mRNA–miRNA–TF), we identified core senescence‐associated genes (Tp53, Cdkn1a, and Myc) as central regulators in myopia progression. Single‐sample gene set enrichment analysis revealed significant immune dysregulation in myopia, marked by altered infiltration of γδ T cells, natural killer T cells, and neutrophils. Functional validation through Tp53 overexpression and Cdkn1a/Myc knockout in mice demonstrated their critical roles in exacerbating myopia phenotypes, including elongated eye axis and thickened retina. These findings highlight a synergistic interplay between cellular senescence and immune‐mediated mechanisms in myopia, supported by multi‐omics evidence and in vivo experiments. Our work not only maps the molecular networks bridging senescence and myopia but also proposes novel therapeutic targets for modulating these pathways. This study advances the understanding of myopia as a senescence‐associated disorder and underscores the potential of targeting immune–senescence crosstalk for intervention.

## Graphical abstract

This study identifies *Tp53*, *Cdkn1a*, and *Myc* as core senescence‐associated regulators driving myopia via immune and apoptotic pathways. Multi‐omics integration and in vivo validation reveal that these genes modulate ocular structure and apoptosis through the NF‐κB axis, highlighting a senescence–immune interplay as a potential therapeutic target in myopia pathogenesis.

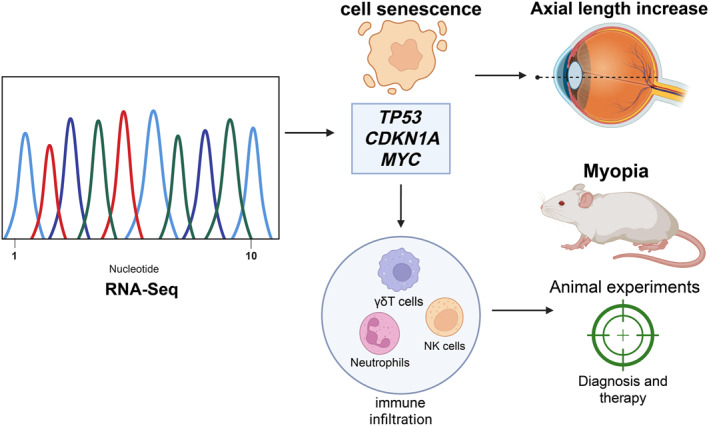

## 1 INTRODUCTION

Myopia is a common eye health problem worldwide, affecting approximately 2.5 billion people globally.^1^, ^2^, ^3^ Myopia is a refractive error primarily caused by the elongation of the eye's axial length. Its etiology is complex, involving the interplay of genetic and environmental factors. In recent years, the prevalence of myopia has been steadily increasing, particularly among adolescents, making it a global public health issue. Although this visual impairment can be temporarily resolved through vision correction, it is associated with an increased risk of severe eye pathologies such as retinal detachment, macular degeneration, and glaucoma, posing a significant public health challenge.^4^ The prevalence of myopia is continuously rising, especially among adolescents. Statistics show that in some regions, the myopia rate among adolescents is as high as over 90%, impacting not only the quality of life of individuals but also increasing the risk of developing other serious eye diseases.

Cellular aging is a stable cell cycle arrest state in which cells enter in response to various sources of stress and is associated with various age‐related diseases.^5^, ^6^ Its hallmark is the secretion of specific phenotypes, including the release of pro‐inflammatory cytokines, growth factors, and extracellular matrix metalloproteinases (MMPs), which can lead to tissue dysfunction. Cellular aging can be triggered by various factors, including DNA damage,^7^ telomere shortening,^8^ oxidative stress, and oncogenic signaling.^9^ Whereas aging cells are beneficial in aspects such as wound healing and tissue remodeling, their prolonged presence in tissues can promote chronic inflammation and mitochondrial dysfunction, leading to conditions such as cancer, diabetes, and neurodegenerative diseases.^10^, ^11^ In myopic patients, age‐related extracellular matrix remodeling and inflammatory signaling may directly influence the progression of myopia by regulating the sclera and axial length of the eye. This concept provides essential insights for exploring the specific molecular mechanisms of cellular senescence in myopia.

The formation of myopia is a complex biological process involving the interplay of genetic and environmental factors. Recent studies suggest a possible association between myopia and cellular aging.^12^ Although a substantial amount of research has uncovered many genetic markers related to myopia, the specific molecular pathways through which these genetic factors affect the physiological structure of the eyes are still under investigation. For instance, aging cells often secrete various MMPs that can remodel the extracellular matrix. In the eyes, the remodeling of the scleral extracellular matrix is a key process in the development of myopia as it influences the shape and refractive power of the eye.^13^

The relationship between cellular aging and myopia is not yet fully understood and is still being studied. However, several pathways suggest that aging may impact the development and progression of myopia: inflammatory signals, cell communication, ocular growth, and development.^14^, ^15^ Although previous studies have revealed a potential link between cellular senescence and myopia, the specific molecular regulatory networks and their relationship with immune‐mediated mechanisms remain unclear.

As a key regulator of ocular tissue, the immune system may influence the expression and regulation of cellular senescence‐related genes through inflammatory responses and immune cell infiltration when dysregulated.^13^ Research has shown that specific immune cells, such as neutrophils and γδ T cells, play a crucial role in ocular pathology, yet their relationship with cellular senescence is poorly understood. Exploring how immune‐mediated mechanisms regulate cellular senescence genes to affect ocular tissue function will provide new insights into the molecular mechanisms of myopia.

To address this research gap, this study employs integrated genomics approaches to systematically analyze the differential expression of cellular senescence‐related genes (CSRGs) and their regulatory networks in myopic samples. This study further elucidates the interaction between cellular senescence and immune mechanisms by combining protein–protein interaction (PPI) networks, mRNA–miRNA interactions, and transcription factor interactions. Specifically, this research investigates the role of immune cell infiltration in myopia and the potential pathways through which cellular senescence influences ocular tissue function via immune mechanisms. Ultimately, through gene knockout and overexpression experiments, the study validates the regulatory roles of these key genes on ocular structure and function in mice, providing new insights into the molecular mechanisms of myopia and offering potential molecular targets for developing future therapeutic strategies.

## 2 MATERIALS AND METHODS

### 2.1 Data acquisition

RNA‐seq datasets, GSE112155^16^ and GSE151631^17^, were retrieved from GEO^18^ via the R package GEOquery (v2.66.0).^19^ GSE112155 includes corneal epithelial samples from 10 myopia and 10 keratitis patients, with only the myopia group used for analysis. GSE151631 comprises count data from 19 keratitis patients and 7 healthy controls, with the control group selected for comparison. Raw counts were converted to TPM, log_2_‐transformed [log_2_(TPM + 1)], and merged. Batch effects were corrected using ComBat from the sva package (v3.44.0)^20^ with empirical Bayes adjustment, followed by between‐sample normalization using normalizeBetweenArrays from limma (v3.52.4). CSRGs were compiled from GeneCards (*n* = 35; search terms: “cellular senescence” and “protein coding”) and CellAge (*n* = 279; via PubMed).^21^ CSRGs were intersected with differentially expressed genes (|logFC| > 1, adjusted *p* values < 0.05) to define cellular senescence‐related DEGs (CSRDEGs). Visualization was performed using ggplot2 (v3.4.0) for volcano plots, pheAtmap (v1.0.12) for heatmaps, and RCircos (v1.2.1) for chromosomal mapping. All analyses used R (v4.2.2).

### 2.2 Differential expression analysis

By merging the count matrices of datasets GSE112155 and GSE151631 and conducting differential analysis using the “DESeq2” software package (V1.36.0),^22^ the DEGs between the myopia group and the normal group were identified. Subsequently, the DEGs with |logFC| > 1 and *p*.adjust < 0.05 were intersected with the CSRGs, and a Venn diagram was created to visualize the overlapping genes, resulting in the identification of the CSRDEGs.

### 2.3 GO and KEGG enrichment analysis

Using the “clusterProfiler” software package,^23^ functional enrichment analysis of the CSRDEGs was performed based on gene ontology (GO)^24^ and Kyoto encyclopedia of genes and genomes (KEGG) pathways (Kanehisa and Goto 2000).^25^ The significance threshold for enrichment was set at *p*.adjust < 0.05 and *q*‐value < 0.05, with the adjustment of *p*‐values performed using the Benjamini–Hochberg (BH) method.

### 2.4 Gene set enrichment analysis

To investigate the biological processes and pathways significantly altered between myopia and control samples, gene set enrichment analysis (GSEA)^26^ was performed using predefined gene sets from the molecular signatures database (MSigDB),^27^ specifically the “c2.cp.v7.2.symbols.gmt” collection. GSEA was conducted in R (v4.2.2) using the clusterProfiler package, with pathway annotation based on the MSigDB C2 curated gene sets. The analysis was performed with 1000 permutations and a random seed set to 2020 to ensure reproducibility. Only gene sets containing between 10 and 500 genes were retained for analysis. Statistical significance was assessed using the Benjamini–Hochberg method for multiple testing correction, with adjusted *p* values < 0.05 and false discovery rate (FDR) *q* values < 0.25 considered significant.

### 2.5 Protein–protein interaction network

The PPI network provides a functional framework for coordinating key biological processes, including signal transduction, gene expression regulation, metabolism, and cell cycle control. To construct the PPI network of candidate CSRDEGs, the STRING database^28^ was employed with a high‐confidence interaction score threshold of 0.700. The resulting network was visualized, and hub genes, defined as CSRDEGs with extensive interactions, were prioritized for further analysis, as they often represent central components of functionally coherent molecular complexes. Key hub genes were identified using the cytoHubba plugin^29^ in Cytoscape by applying five topological algorithms: degree, maximum neighborhood component (MNC), maximum clique centrality (MCC), edge percolated component (EPC), and closeness. The top 10 genes ranked by each method were intersected to determine central candidates. Additionally, GeneMANIA^30^ was used to predict functionally related genes and construct an extended interaction network for the identified CSRDEGs.

### 2.6 Construction of mRNA–miRNA and mRNA–TF interaction networks

The miRDB database^31^ provides tools for predicting microRNA (miRNA) target genes and functional annotations. We utilized miRDB to predict miRNAs that interact with the identified hub genes, selecting miRNAs with target scores ≥85 to construct an mRNA–miRNA interaction network. Furthermore, the CHIPBase database v3.0^32^ determines binding motifs and TF regulatory relationships through the analysis of ChIP‐seq data. We used CHIPBase to search for TFs that bind to the hub genes, generating an mRNA–TF interaction network with a threshold of the sum of discovered samples (upstream) + discovered samples (downstream) ≥5.

### 2.7 Group comparison analysis and ROC curve

The receiver operating characteristic (ROC) curve^33^ is a graphical tool for evaluating the diagnostic performance of biomarkers by depicting the trade‐off between sensitivity and specificity. The area under the curve (AUC) ranges from 0.5 to 1.0, with higher values indicating greater diagnostic accuracy. To assess the diagnostic potential of hub gene expression in myopia, ROC curves were generated using the pROC package (v1.18.0), and AUC values were calculated for each hub gene across normal and myopia groups.

### 2.8 Immune infiltration analysis

Single‐sample gene set enrichment analysis (ssGSEA)^34^ was employed to estimate the relative abundance of immune cell infiltration in each sample. A curated set of human immune cell subtypes was used, including activated CD8^+^ T cells, activated dendritic cells, γδ T cells, natural killer (NK) cells, and regulatory T cells. ssGSEA enrichment scores were calculated to generate an immune cell infiltration matrix, representing the relative abundance of each immune cell type across samples. Group‐wise comparisons of ssGSEA scores were visualized using ggplot2 to highlight differences in immune cell distributions between conditions. A correlation heatmap was generated with pheAtmap to display the relationships among immune cell types, followed by correlation analysis between hub gene expression and immune cell infiltration levels to identify significant immune–gene associations within the dataset.

### 2.9 Establishment of CRISPR/Cas9 gene knockout mouse model

Specific sgRNA sequences targeting Tp53, Cdkn1a, and Myc were designed using the online tool CRISPOR. The sequences were as follows: Tp53 (F: AGGACTCGGCGCGCCGGAAG TGG, R: GGCCTAAGGACTCGGCGCGC CGG), Cdkn1a (F: CCGCGACTGTGATGCGCTAA TGG, R: CCATTAGCGCATCACAGTCG CGG), and Myc (F: GCCGTATTTCTACTGCGACG AGG, R: GAAGGGTGTGACCGCAACGT AGG). The designed sgRNAs were cloned into a Cas9‐expressing plasmid vector using standard molecular cloning techniques, including PCR amplification (Cat# 25745, ABC Biotech), restriction digestion (Cat# 58642, XYZ Diagnostics), and ligation (Cat# 55763, 123 Genomics). The constructed sgRNA‐Cas9 expression vectors were microinjected into mouse zygotes under sterile conditions by trained personnel to ensure embryo viability and experimental success. Microinjection was performed using an injection system (Cat# 77852, Injection Systems Co.) and glass capillary needles (Cat# 66574, Needles R Us). Embryos were implanted into pseudopregnant C57BL/6 female mice (Cat# 55847, Lab Animals Ltd.), following institutional animal ethics and welfare guidelines. After birth, genomic DNA was extracted from the tail tissue, and gene knockout was confirmed by PCR and Sanger sequencing. Mice were assigned to four groups: control, Tp53 knockout, Cdkn1a knockout, and Myc knockout.

### 2.10 Lentiviral overexpression

The cDNA sequences of Tp53, Cdkn1a, and Myc were cloned into pLVX‐zsGreen‐C1 using Phusion polymerase (Thermo Fisher) and EcoRI/NotI restriction enzymes (NEB). Lentiviral particles were produced by co‐transfecting HEK293T cells with the expression vector and packaging plasmids using Lipofectamine 3000 (Thermo Fisher). Supernatants were collected at 48–72 h, concentrated via ultracentrifugation, and titrated by limiting dilution. For intraocular delivery, mice were anesthetized with isoflurane (Baxter) and treated with 0.5% proparacaine drops (Alcon). Lentivirus was microinjected into the eye, followed by ibuprofen (Pfizer) analgesia for 48 h. Post‐injection, ocular tissues were harvested under anesthesia. RT‐qPCR and Western blot confirmed overexpression efficacy. All procedures complied with animal welfare guidelines (Approval No. LYEYEC2023‐039).

Tp53: F: 5′ GTCTCCCTCGTCCTCTGC 3/R: 5′ ACTCAACCGTTAGCTCCG 3′
Cdkn1a: F: 5′ GAACCGGCTGGGGATGTC 3′/R: 5′ CCGTTTTCGACCCTGAGA 3′
Myc: F:5′ CTGGCAAAAGGTCAGAGT 3′/5′ TTGTGTTTCAACTGTTCTCGTCGTT 3′

### 2.11 Measurement of ocular morphology

Before the measurements, mice are placed in a dark‐adapted state for approximately 1 h to stabilize their pupil status. Before the measurements, 0.5% proparacaine eye drops (PRCAIN, USA Alcon) are used as a local anesthetic to alleviate discomfort during the measurement process. We used a high‐resolution optical coherence tomography (OCT) device (Cat. No: 57486, Heidelberg Engineering) to regularly examine the ocular morphology of the gene knockout mice, overexpression mice, and normal control group mice. Before performing the OCT scan, mice need to adapt to the dark for 30 min to naturally dilate their pupils and ensure clear imaging. The use of 0.5% proparacaine eye drops as a local anesthetic helps reduce discomfort in mice during the examination. Through OCT scanning, we meticulously recorded and analyzed the axial length and retinal thickness of the mice.

### 2.12 Measurement of refractive status

Before the measurements, mice are placed in a dark‐adapted state for approximately 1 h to stabilize their pupil status. Prior to measurements, 0.5% proparacaine local anesthetic eye drops (US Alcon, Cat No: 123456) are administered to reduce discomfort during the measurement process. Compound tropicamide eye drops (Santen, Cat No: 789101) are used to dilate each mouse's eyes, administered every 5 min for a total of three times. The testing is conducted once the pupils of the mice are fully dilated. During the testing, the mice are secured using a specialized animal fixation device (Stoelting, Cat No: 654321) to ensure the stability of their heads. The position of the auto refractometer (Topcon, Cat No: 987654) is adjusted to align with the mice's eyes. The refractive power is automatically observed and adjusted through the monitoring system to ensure the light spot accurately falls on the fundus of the mouse's eye. The refractive power readings displayed by the refractometer, including astigmatism and axis, are recorded. The refractive status of each mouse is measured once a week and continuously recorded for 4–6 weeks to analyze the trend in refractive status changes, monitoring the progression of myopia.

### 2.13 Ocular physiological function testing

Using a slit lamp microscope (BQ900, Haag‐Streit, serial number DEF456), we conducted a detailed examination of the anterior segment structures of the mouse eye, including the cornea, iris, and lens. Prior to the slit lamp examination, the mice were lightly anesthetized to reduce stress and discomfort. The anesthetic used was 0.5% proparacaine eye drops (Alcon, serial number 123456) to ensure the safety and stability of the examination process. Eye pressure measurements were taken using a Tonopen tonometer (Reichert, serial number GHI789). This method is quick, accurate, and minimally invasive for the mice. Before measuring the intraocular pressure, local anesthesia was applied with 0.5% proparacaine to reduce the discomfort of the mice. The eye pressure of each mouse was measured three times consecutively, and the average value was taken to ensure the accuracy of the data. All examination data were recorded in a dedicated laboratory record sheet, including detailed descriptions of the slit lamp examination and specific values of intraocular pressure.

### 2.14 qRT‐PCR

Total RNA is extracted from cultured cells using TRIzol reagent (Magen). The RNA (1 μg) is reverse transcribed into cDNA using a reverse transcription system (A3500, Promega) with a reaction volume of 10 μL. Oligo(dT)15 primers (C110A, Promega) and random primers (C118A, Promega) are used as the reverse primers. The reverse products are obtained using a 2× RealStar Green Power Mixture (GenStar) in the LightCycler 480 Real‐Time PCR System (LC480, Roche). GAPDH is used as the housekeeping gene. The relative expression level of the target gene is calculated using the 2^−ΔΔCt^ method, and normalization is done using the average Ct value of GAPDH.

Tp53: F:5′ CTTTGAGGTGCGTGTTTG 3′/R: 5′ AGTGGTTTCTTCTTTGGC 3′
Cdkn1a: F: 5′ TGAGTTGGGAGGAGGCAG 3′/R: 5′ GAGCGAGGCACAAGGGTA 3′
Myc: F: 5′ GTCTTCCCCTACCCTCTC 3′/R: 5′ TCCTCATCTTCTTGTTCC 3′
Bcl‐2: F: 5′ GTGTTCGGTGTAACTAAAGAC 3′/R: 5′ AGCTAGCTCACTTGTGGCCCAGGTATG 3′
Bax: F: 5′ AAGCTGAGCGAGTGTCTCCGGCG′/R: 5′ GCCACAAAGATGGTCACTGTCTGCC 3′
P53: F: 5′ CCTCCCCAGCATCGTATC 3′/R: 5′ CCTCGGGTGGCTCATAAG 3′
Rip1: F: 5′ CTGAAAGAGAACAACGAC 3′/R: 5′ TTTTACCTAATGGAATGG 3′
Caspase‐8: F: 5′ GTGAAGAACTGCGTTTCCTACC 3′/R: 5′ AGCTTCTTCCGTAGTGTGAAGG 3′
Traf2: F: 5′ CAGCAGGTACGGCTACAA 3′/R: 5′ GCCCTTCATCACCACAAA 3′
Glut1 (Slc2a1): F: 5′‐AGCCCTGCTACAGTGTAT‐3′/R: 5′‐AGGTCTCGGGTCACATC‐3′
Cyclin D1 (Ccnd1): F: 5′‐GCGTACCCTGACACCAATCTC‐3′/R: 5′‐CTCCTCTTCGCACTTCTGCTC‐3′

### 2.15 Western blot

The samples are lysed in cell lysis buffer containing phenylmethylsulfonyl fluoride (Beotime) and phosphatase inhibitor (Beotime). The cell lysates are then subjected to 10%–15% sodium dodecyl sulfate‐polyacrylamide gel electrophoresis and semi‐dry transferred onto a polyvinylidene fluoride membrane (Roche). The membrane is blocked with 3% bovine serum albumin (Reshu) in Tris‐buffered saline with Tween 20 (TBST, 20 mM Tris‐HCl pH 8.0, 150 mM NaCl, and 0.05% Tween 20) for 1 h at room temperature. After blocking, the membrane is incubated with the designated primary antibody overnight at 4°C. Subsequently, the membrane is probed with the corresponding secondary antibody.

### 2.16 Hematoxylin and eosin staining

The eye tissue samples are sectioned into appropriate tissue blocks and fixed in 4% neutral buffered formalin (Reshu) for 18 h. The tissue pathology sections are stained using hematoxylin and eosin (H&E) staining (Antarctic Ketai Company) following the manufacturer's instructions.

### 2.17 Immunohistochemistry

The tissue sections are placed on glass slides coated with silane, decarbonated, and rehydrated in low‐concentration ethanol. Subsequently, the sections are treated with cell and tissue staining kit reagents (CTS005, CTS002, R&D) following the manufacturer's instructions and incubated in a 10 mM citrate buffer for heat‐induced antigen retrieval. The concentrations of the primary antibodies used are: rabbit anti‐Tp53 (1:2000, GTX129270, GeneTex), rabbit anti‐Cdkn1a (Asp297) (1:2000, 4199s, CST), and mouse anti‐Myc (1:1000, CST).

### 2.18 Genetic manipulation

Human retinal pigment epithelial cells (ARPE‐19) are cultured in 10% fetal bovine serum, Dulbecco's modified eagle medium (DMEM) at 37°C with 5% CO_2_ to observe and maintain regular cell growth. The Tp53, Cdkn1a, and Myc genes are silenced using small interfering RNA (siRNA) technology. siRNA sequences targeting these genes are designed and introduced into cells using a transfection reagent. The siRNA is diluted in 25 μL DMEM medium without antibiotics and serum, with 1.25 μL of siRNA (stored at 20 μM/L) added for a final concentration of 50 nM. 1.0 μL of EpFed transfection reagent is added to the mixture, gently flicked 3–5 times with a pipette to mix, and then incubated at room temperature for 20 min. The transfection mixture is evenly added to the cells in a 24‐well plate, gently rocking the plate back and forth to ensure thorough mixing. The expression levels of the genes and proteins are detected using qPCR and Western Blot to confirm the efficacy of the gene manipulation. Cells were treated with the specific activator TNF‐α (MCE, HY‐P1860) to induce activation of the NF‐κB signaling pathway, following the manufacturer's instructions for treatment conditions.

### 2.19 Cell cycle analysis

The cells are seeded into cell culture plates and cultured in a DMEM medium (Gibco, Cat No 11965‐092). After digestion with trypsin (Gibco, Cat No 25200‐056), the cells are gently pipetted to avoid cell damage and adhesion. The supernatant is centrifuged (1500 rpm, 4 min) and washed 2–3 times with pre‐chilled PBS (Gibco, No. 10010‐023) to remove the DMEM medium, residual drugs, and cell debris. A small amount of PBS is added to the cell pellet; then, the cells are gently resuspended. The suspended cells are then fixed in pre‐chilled 70% ice‐cold ethanol (Sigma‐Aldrich, catalog number E7023), gently mixed, sealed with plastic wrap, and stored at 4°C overnight. After that, the cells are centrifuged at ≈450 × *g* for 4 min; the supernatant is removed, washed twice with PBS, and then 100 μL of 100 μg/mL RNase A (Qiagen, No. 19101) and 0.2% Triton X‐100 (Sigma‐Aldrich, No. T8787) are suspended at 37°C for 30 min. This is followed by adding 400 μL of 50 μg/mL PI (Sigma‐Aldrich, No. P4170), mixing well, and incubating at room temperature in the dark for 30 min. The cell cycle is then analyzed using a flow cytometer (BD FACSCalibur, BD Biosciences, No. 342975), counting 100,000 cells, detecting red fluorescence at an excitation wavelength of 488 nm, and analyzing the cell cycle phase distribution using FlowJo software (BD Biosciences, No. 00‐0000‐00) using FL2‐w and FL2‐A display, to remove clumped cells. During the machine detection, the cell suspension must be thoroughly resuspended to avoid clogging the instrument's tubing; if there are too many cells or severe clumping, they can be filtered through a 300 mesh (aperture 40–50 μm) nylon mesh (Millipore, No. NY3002500) before machine detection. Flow cytometry is used to analyze the cell cycle status of genetically manipulated cells, with cells labeled with PI dye for analysis of different cell cycle phases.

### 2.20 Cell apoptosis detection

After transfection for 24–72 h, cells are washed with PBS. The cells are then treated with trypsin or an enzyme‐free cell dissociation buffer to collect a cell suspension. The collected cells are resuspended in PBS to a cell concentration of 1 × 10^6^ cells/mL. Annexin V and PI dyes are added to the cell suspension, and staining is carried out according to the kit instructions. The stained cells are analyzed using a flow cytometer, and appropriate fluorescence channels are set to detect Annexin V and PI signals. Cell apoptosis stages are analyzed through software: live cells (Annexin V^−^PI^−^), early apoptotic cells (Annexin V^+^PI^−^), and late apoptotic or dead cells (Annexin V^+^PI^+^).

### 2.21 Cell proliferation experiment

After an appropriate cell culture period (usually 24–72 h), add 10 μL of CCK‐8 reagent (Dojindo, Japan, Cat No: CK04) into each well. Incubate: Place the 96‐well plate in the incubator for 1–4 h to allow the CCK‐8 reaction to occur. Measure the optical density (OD) of the plate at 450 nm wavelength using a BioTek instrument (Model: ELx800). The OD value is directly proportional to the activity and quantity of the cells.

### 2.22 Laboratory animal ethical statement

The license number for this research proposal is 2021 × 124, approved by the Animal Ethics Committee of the Second Hospital of Longyan City. Animal experiments were conducted by following the “Guidelines for the Care and Use of Laboratory Animals” and the “Laboratory Animals—Environmental and Husbandry Facilities Requirements” (GB18712‐2015/XG1‐2024, National Standardization Technical Committee for Laboratory Animals) to ensure humane treatment and ethical standards for animals.

### 2.23 Statistical analysis

All data processing and statistical analysis were performed using R software. Continuous variables are presented as mean ± standard deviation. The comparison between two groups of continuous variables was conducted using the Wilcoxon rank‐sum test, and the statistical significance of normally distributed variables was assessed using the independent Student's *t*‐test. For comparisons involving three or more groups, the Kruskal–Wallis test was employed. Comparisons between two groups of categorical variables were conducted using either the Chi‐squared test or Fisher's exact test, depending on the situation. ROC curve analysis was used to evaluate the diagnostic performance of central genes, and Spearman correlation analysis was used for correlation testing. All tests were two‐tailed, and a *p* value of <0.05 was considered statistically significant.

## 3 RESULTS

### 3.1 GEO data integration and analysis reveal the potential role of CSRGs in myopia

**FIGURE 1:**
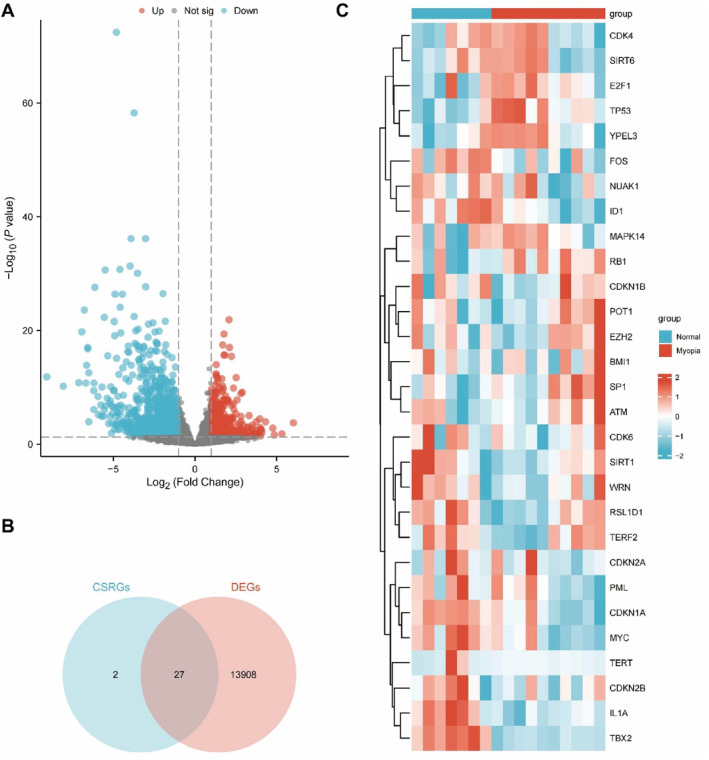
Differential analysis between normal and myopia groups. (A) Volcano plot depicting the DEGs between the normal and myopia groups in the merged dataset. (B) Venn diagram showing the overlap of DEGs and CSRGs in the merged dataset. (C) Heat map of CSRDEGs in the merged dataset. CSRDEGs, cellular senescence‐related DEGs; CSRGs, cellular senescence‐related genes; DEGs, differentially expressed genes.

A comprehensive analysis of count‐based sequencing data from datasets GSE112155 and GSE151631 was performed, followed by normalization to TPM. As shown in Figure S1A,B, the selected samples formed a unified dataset, indicating that batch effect correction and normalization effectively minimized inter‐sample variability. Differential expression analysis of the merged data identified 13,935 DEGs, as visualized in a volcano plot (Figure 1A). To identify CSRDEGs, known CSRGs were intersected with the DEGs, and the overlap was presented in a Venn diagram (Figure 1B). A total of 29 CSRDEGs were identified, including ATM, BMI1, CDK6, CDK4, CDKN1A, CDKN1B, CDKN2A, CDKN2B, E2F1, EZH2, FOS, ID1, IL1A, MAPK14, MYC, NUAK1, PML, POT1, RB1, RSL1D1, SIRT1, SIRT6, SP1, TERF2, TP53, WRN, and YPEL3. The expression patterns of these genes across samples were visualized using a heatmap (Figure 1C).

### 3.2 Enrichment analysis reveals the potential functions and pathways of cellular senescence‐related genes in myopia

**FIGURE 2:**
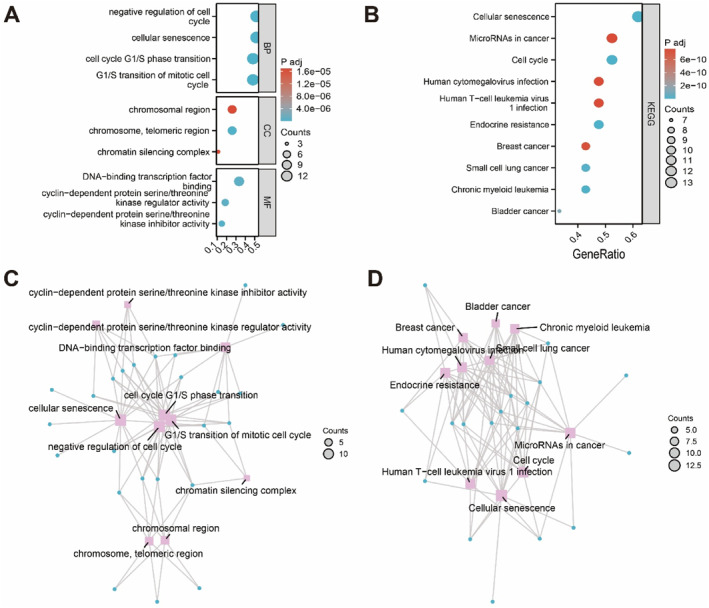
GO and KEGG enrichment analysis. (A) GO enrichment of biological processes, cellular components, and molecular functions. (B) KEGG pathways associated with CSRDEGs. (C) Network of CSRDEGs and enriched GO terms. (D) Network of CSRDEGs and KEGG pathways. CSRDEGs, cellular senescence‐related DEGs; GO, gene ontology; KEGG, Kyoto encyclopedia of genes and genomes.

To characterize the functional roles of the 27 CSRDEGs in the context of myopia, GO and KEGG enrichment analyses were performed (FDR < 0.05). GO analysis revealed significant enrichment of CSRDEGs in biological processes such as cellular aging and cell cycle G1/S phase transition; in cellular components such as chromosome regions, telomere regions, and chromatin silencing complexes; and in molecular functions including DNA‐binding transcription factor activity and serine/threonine kinase inhibitor activity related to cell cycle regulation (Figure 2A). KEGG pathway analysis indicated that CSRDEGs were enriched in pathways associated with cellular senescence, cell cycle control, human cytomegalovirus infection, human T‐cell leukemia virus infection, insulin resistance, and multiple cancer types (Figure 2B). The relationships between CSRDEGs and enriched GO terms and KEGG pathways were visualized using network diagrams, illustrating the complexity of gene–function and gene–pathway associations (Figure 2C,D). These results highlight the multifaceted biological roles of CSRDEGs and their potential involvement in myopia‐related processes.

### 3.3 PPI network reveals key senescence‐related genes in the pathogenesis of myopia

**FIGURE 3:**
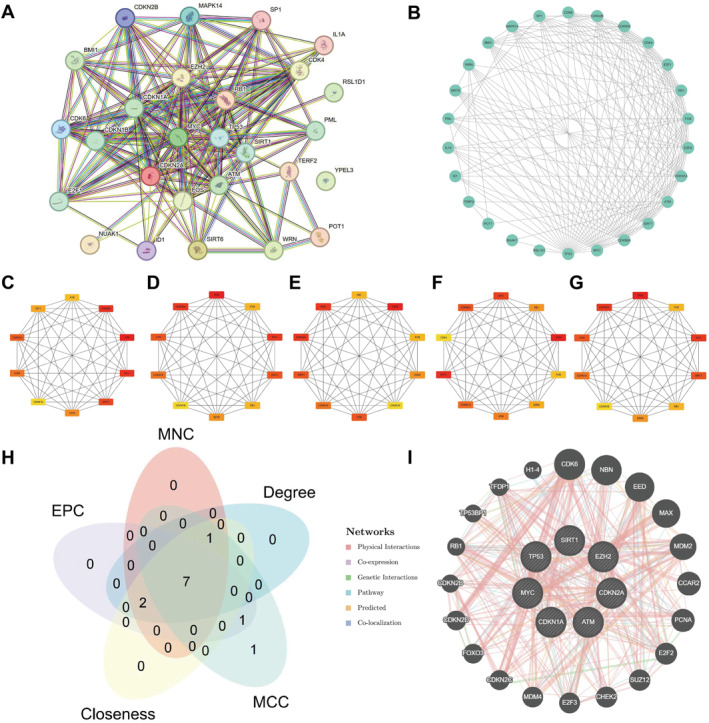
PPI network of hub genes. (A) PPI network from the STRING database. (B) Visualization of the PPI network using Cytoscape software. (C–G) PPI network of hub genes analyzed using algorithms: MCC (C), closeness (D), degree (E), EPC (F), and MNC (G), with color gradient indicating scores from low to high. (H) Venn diagram displaying the intersection of top‐ranked central genes based on closeness, degree, EPC, MCC, and MNC algorithms. (I) Interaction network of the seven central genes. EPC, edge percolated 139 component; MCC, maximum clique centrality; MNC, maximum 138 neighborhood component; PPI, protein–protein interaction.

PPI analysis of the 29 CSRDEGs was performed using the STRING database, and the interaction network was visualized with Cytoscape software (Figure 3A,B). Among them, 26 CSRDEGs exhibited interactions with other genes. Further analysis using the cytoHubba plugin revealed centrality scores for these genes across five algorithms—MCC (Figure 3C), closeness (Figure 3D), degree (Figure 3E), EPC (Figure 3F), and MNC (Figure 3G). Centrality scores for all 26 genes across the five algorithms are provided in Table S1. By intersecting the top 10 ranked genes from each algorithm (Figure 3H), seven key hub genes were identified: ATM, EZH2, CDKN1A, CDKN2A, MYC, SIRT1, and TP53. In addition, functional interaction networks for these hub genes were predicted and constructed using the GeneMANIA platform, revealing co‐expression, co‐localization, and physical interactions among them (Figure 3I).

### 3.4 mRNA–miRNA and mRNA–TF interaction networks reveal the regulatory mechanisms of myopia‐related genes

MicroRNAs play essential regulatory roles in various biological processes, particularly in cell cycle control, senescence, and immune responses. To investigate the regulatory potential of myopia‐associated senescence genes, miRNA–mRNA interactions were predicted using the miRDB database. A high‐confidence interaction network (score >90) was constructed for seven hub genes (ATM, EZH2, CDKN1A, CDKN2A, MYC, SIRT1, and TP53), revealing a complex mRNA–miRNA regulatory landscape (Figure S2A). The network shows that multiple miRNAs target more than one hub gene, suggesting potential coordinated regulation. In parallel, transcriptional regulation was explored using the hTFtarget database. A total of 155 mRNA–TF interaction pairs were identified for six of the seven hub genes, forming a transcription factor regulatory network (Figure S2B). These findings suggest that transcription factors may indirectly contribute to ocular homeostasis and myopia by regulating senescence‐related genes. Although functional validation is pending, the constructed regulatory networks provide potential targets and a systems‐level framework for future mechanistic studies.

### 3.5 Comparative analysis of central gene expression differences and immune cell infiltration patterns in myopia reveals immune mechanisms in myopia

**FIGURE 4:**
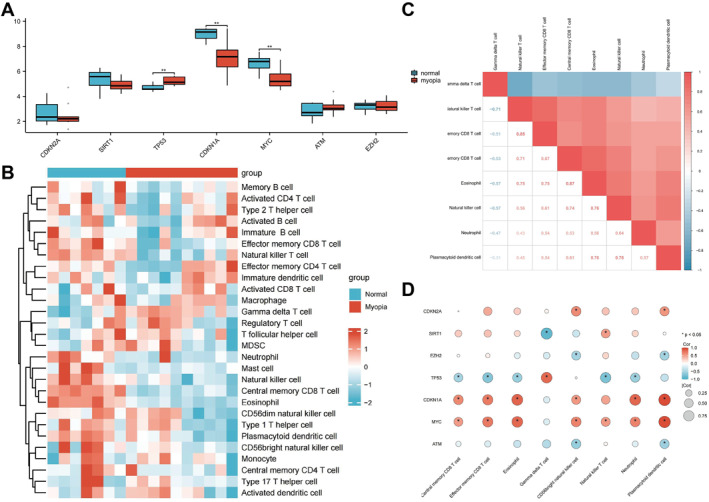
Comparative analysis of key gene expression and immune cell infiltration patterns between myopia and normal vision groups. (A) Group comparison plot depicts the expression differences of central genes in the dataset. (B) Heat map displaying the ssGSEA immune cell infiltration analysis results in the dataset samples. (C) Results of correlation analysis show the abundance of immune infiltration in the dataset samples. (D) Heat map illustrating the correlation between immune cell infiltration abundance and expression levels of the seven central genes in the dataset samples. Significance levels are denoted as follows: *p ≤ 0.05; **p ≤ 0.01; and ns: *p* > 0.05. ssGSEA, single‐sample gene set enrichment analysis.

Mann–Whitney *U* tests were performed to assess the differential expression of hub genes between the normal and myopia groups. Significant differences were observed for Tp53, Cdkn1a, and Myc (*p* < 0.01), as shown in the comparative plot (Figure 4A). The predictive performance of these three genes was further evaluated using ROC analysis, with AUC values ranging from 0.8 to 0.9, indicating moderate diagnostic accuracy (Figure S3A).

To assess immune cell infiltration, the relative abundance of 28 immune cell types was quantified using the ssGSEA algorithm. A heatmap illustrated overall infiltration patterns (Figure S3B), and Mann–Whitney *U* tests revealed significant differences in eight immune cell types between the two groups (*p* < 0.05), including γδ T cells, natural killer T (NKT) cells, effector memory CD8 T cells, central memory CD8 T cells, eosinophils, natural killer cells, neutrophils, and plasmacytoid dendritic cells (Figure 4B). Correlation analysis showed a negative association between γδ T cells and other cell types, whereas positive correlations were observed among the remaining cells, with the strongest correlation between eosinophils and central memory CD8 T cells (Figure 4C).

The correlation between hub gene expression (ATM, EZH2, CDKN1A, CDKN2A, MYC, SIRT1, and TP53) and the infiltration levels of the eight immune cell types was also examined. Significant associations (*p* < 0.05) were visualized in a correlation heatmap V, highlighting interactions between gene expression and immune cell infiltration patterns (Figure 4D).

### 3.6 The impact of aging gene knockout or overexpression on mouse eye growth

**FIGURE 5:**
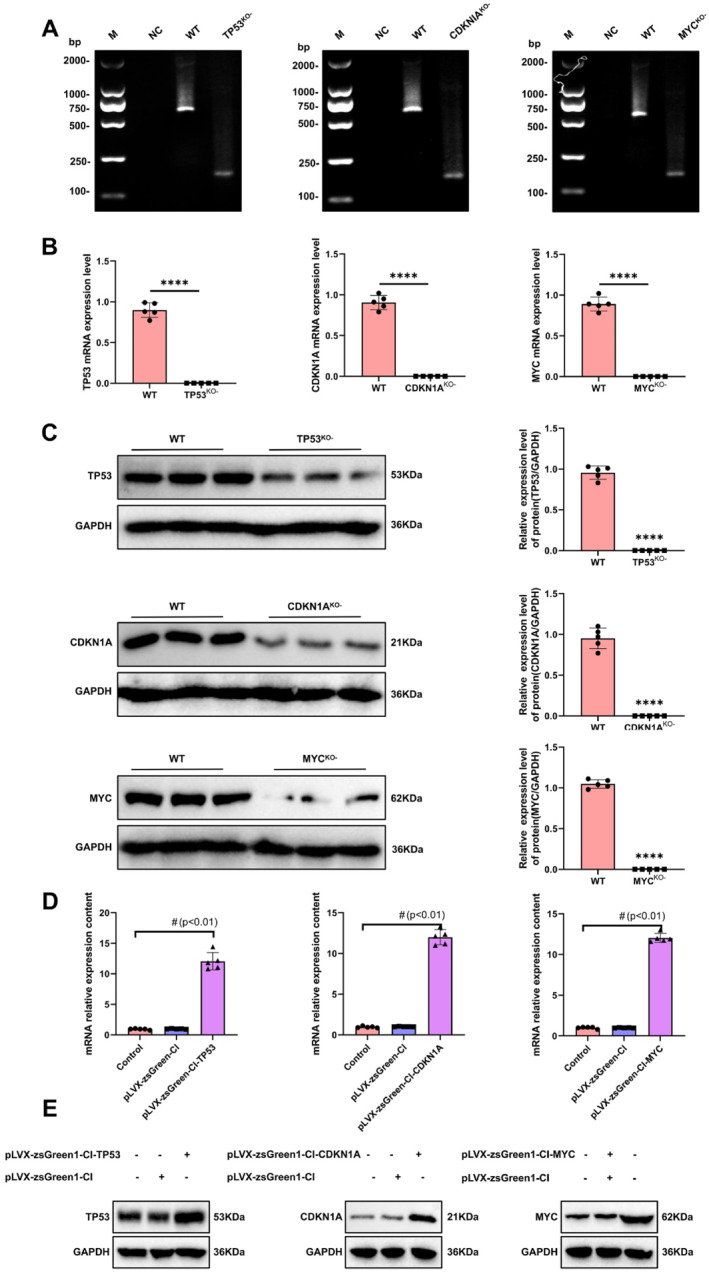
Validation of gene knockout and overexpression mouse models. (A) PCR detection of Tp53, Cdkn1a, and Myc gene knockout in mouse retinal tissues. M, marker; NC, negative control; KO, gene knockout type; and WT, wild type. Each group has 3 samples. (B) qPCR analysis of mRNA expression levels of Tp53, Cdkn1a, and Myc genes in WT and knockout mouse retinal tissues. Data are presented as mean ± SEM, with 5 samples. (C) Western blot analysis of protein expression levels of Tp53, Cdkn1a, and Myc in WT and knockout mouse retinal tissues. GAPDH is used as an internal control, and data are presented as mean ± SEM with 3 samples. (D) qPCR analysis of mRNA expression levels of Tp53, Cdkn1a, and Myc genes in mouse cells transfected with overexpression vectors. The control group consists of untransfected cells, and pLVX‐zsGreen1 is the empty vector control. Data are presented as mean ± SEM, with 3 samples. (E) Western blot analysis of protein expression levels of Tp53, Cdkn1a, and Myc in mouse cells transfected with overexpression vectors. The control group consists of untransfected cells, pLVX‐zsGreen1 is the empty vector control, and GAPDH is used as an internal control. Statistical analysis was performed using a *t*‐test, with significance denoted as follows: ****p < 0.0001 and ^#^ *p* < 0.01. qPCR, quantitative PCR; SEM, standard error of the mean.

Gene knockout mouse models for Tp53^−/−^, Cdkn1a^−/−^, and Myc^−/−^ displayed shorter DNA bands compared to wild‐type controls, indicating successful gene deletion (Figure 5A). Correspondingly, qPCR and Western blot analyses showed that mRNA and protein levels of the target genes were nearly undetectable in knockout mice (Figure 5B,C). In mice injected intraocularly with lentiviral vectors overexpressing Tp53, Cdkn1a, or Myc, retinal cells exhibited markedly elevated mRNA expression levels compared to controls (Figure 5D), and this was validated at the protein level by Western blotting (Figure 5E), consistent with the transcriptional changes.

**FIGURE 6:**
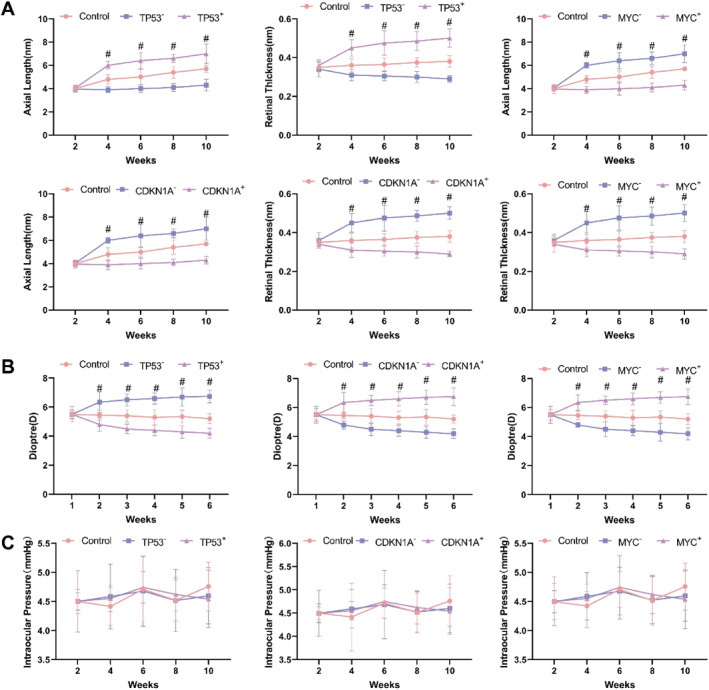
Impact of Tp53, Cdkn1a, and Myc on ocular parameters in mice. (A) Changes in axial length and retinal thickness in mice. (B) Curve depicting changes in mouse refractive error. (C) Assessment of ocular physiological function in mice. Statistical analysis was performed using a *t*‐test, with data significance indicated as follows: ^#^ *p* < 0.01.

Axial length measurements revealed a gradual increase over time in control mice. More pronounced axial elongation was observed in the Tp53 overexpression, Cdkn1a^−/−^, and Myc^−/−^ groups, whereas Tp53^−/−^, Cdkn1a overexpression, and Myc overexpression groups showed a slower rate of axial elongation (Figure 6A). Retinal thickness also increased more significantly over time in the Tp53^−/−^, Cdkn1a overexpression, and Myc overexpression groups compared to controls, although this thickening trend gradually diminished (Figure 6A). Refractive error analysis showed a progressive myopic shift in the Tp53 overexpression, Cdkn1a^−/−^, and Myc^−/−^ groups, while Tp53^−/−^, Cdkn1a overexpression, and Myc overexpression groups exhibited a hyperopic shift over time (Figure 6B).

Assessment of ocular structures showed no notable abnormalities in the control group. In the Tp53 overexpression, Cdkn1a^−/−^, and Myc^−/−^ groups, thinning of the cornea and thickening of the lens were observed, with no significant changes in the iris. Overexpression groups displayed no apparent structural abnormalities (Figure 6C). Intraocular pressure remained consistent across all groups, suggesting that the effects of Tp53, Cdkn1a, and Myc on myopia development are independent of intraocular pressure (Figure 6C).

### 3.7 Aging genes can regulate cell apoptosis

**FIGURE 7:**
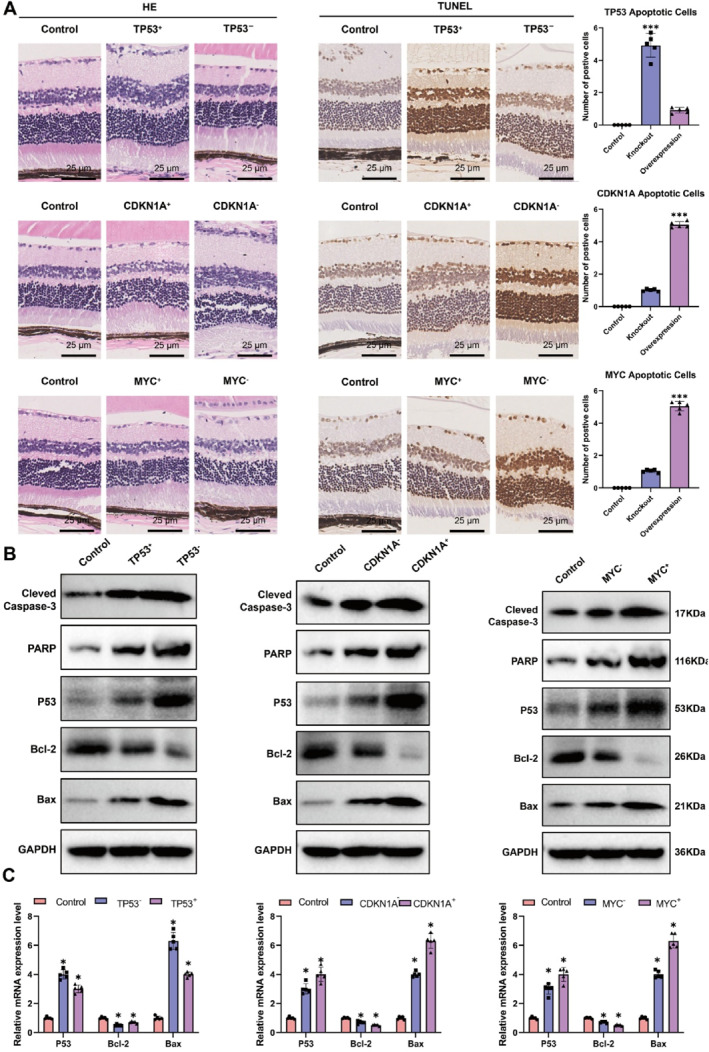
Induction of cell apoptosis in mice through gene knockout or overexpression. (A) Impact of gene knockout or overexpression on ocular tissues in mice. Detection of apoptotic cells in the mouse retina. (B) Detection of apoptotic proteins in mouse cells. (C) Measurement of mRNA levels of apoptotic proteins in mouse cells. Significance levels are denoted as follows: *p ≤ 0.05; ***p ≤ 0.001.

To more directly observe the effects of aging genes Tp53, Cdkn1a, and Myc knockout and overexpression on mouse eyeballs, we prepared H&E sections of mouse eyeballs. Compared to the control group, the sections of mouse eyeballs from the Tp53 overexpression group, Cdkn1a gene knockout group, and Myc gene knockout group showed increased thickness and enlarged gaps between the retina and retinal epithelium. The eyeball cells appeared loose. Additionally, in comparison to the control group, the collagen fibers at the posterior pole of the sclera in the Tp53 gene knockout group, Cdkn1a overexpression group, and Myc overexpression group were disordered and thin, with uneven thickness between collagen fibers, enlarged gaps, and nuclear deformation (Figure 7A).

Furthermore, to explore the relationship between aging genes and cell apoptosis in myopia, we examined the apoptosis status of retinal cells in each experimental group of mice. No positive apoptotic nuclei were found in the retinas of the control group mice. In comparison to the control group, the Tp53 knockout group, Cdkn1a overexpression group, and Myc overexpression group exhibited more apoptotic positive nuclei, mainly expressed in the ganglion cell layer, with a small amount expressed in the nuclear layer. Additionally, in comparison to the control group, the Tp53 overexpression group, the Cdkn1a gene knockout group, and the Myc gene knockout group of mice showed a small number of positive apoptotic nuclei expressed in both the ganglion cell layer and the nuclear layer of the retina (Figure 7A).

Moreover, Western Blot validation was performed on the relevant proteins in the remaining eye tissues of each group of mice, and the experimental results indicated apoptosis in the eye tissues of both gene knockout and overexpression groups. However, the degree of apoptosis in the Tp53 overexpression group, Cdkn1a gene knockout group, and Myc gene knockout group was lower compared to the Tp53 gene knockout group, Cdkn1a gene knockout group, and Myc gene knockout group (Figure 7B). Additionally, the apoptosis status of each experimental group was validated at the mRNA level, with the trends consistent with the protein results (Figure 7C).

### 3.8 The impact of aging genes on cellular biological processes

**FIGURE 8:**
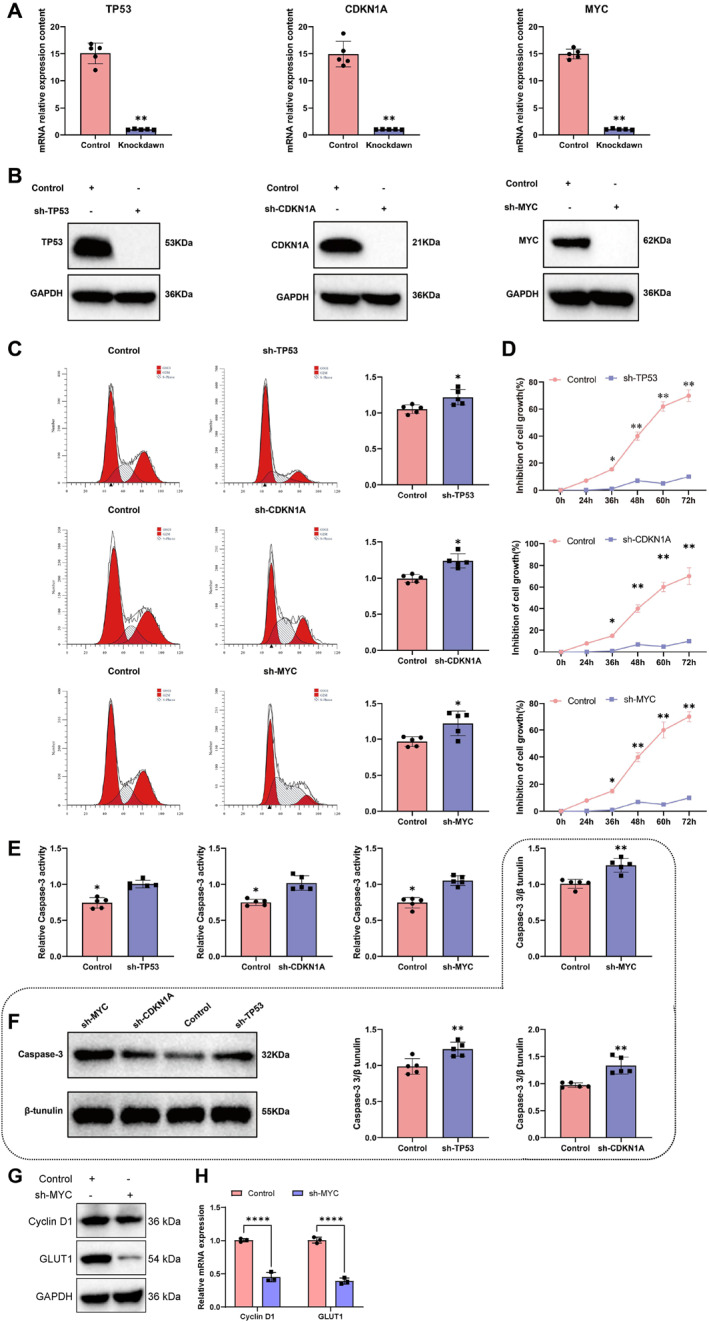
Impact of aging genes on cellular biological processes. (A) Validation of gene siRNA transfection in reducing cellular mRNA levels. (B) Validation of gene siRNA transfection in reducing cellular protein levels. (C) Changes in the cell cycle post gene manipulation. (D) Changes in cell viability post gene manipulation. (E) Western blot analysis of Cyclin D1 and GLUT1 protein expression, downstream targets of Myc. (F) RT‐qPCR analysis of Cyclin D1 and GLUT1 mRNA expression, downstream targets of Myc. (G) Changes in Caspase‐3 activity following gene manipulation. (H) Caspase‐3 protein levels after gene manipulation. Significance levels are denoted as follows: *p ≤ 0.05; **p ≤ 0.01; ****p ≤ 0.001; ns: *p* > 0.05. siRNA, small interfering RNA.

Following siRNA‐mediated knockdown of Tp53, Cdkn1a, and Myc in ARPE‐19 cells, both qPCR and Western blot analyses confirmed a significant reduction in mRNA and protein expression levels compared to controls (Figure 8A,B). Flow cytometry revealed that Tp53 knockdown led to cell cycle prolongation, whereas Cdkn1a and Myc knockdown resulted in shortened cell cycles (Figure 8C). CCK‐8 assays demonstrated that silencing any of the three genes significantly inhibited cell proliferation (Figure 8D).

Additionally, knockdown of Myc led to marked downregulation of its classical transcriptional targets Cyclin D1 and Glut1, as shown by both qPCR and Western blot analysis (Figure 8E,F). Caspase‐3 assays indicated increased enzymatic activity (Figure 8G) and elevated protein expression levels (Figure 8H) in all knockdown groups, consistent with in vivo results. These findings highlight the essential roles of Tp53, Cdkn1a, and Myc in regulating cell cycle progression, proliferation, and apoptosis.

### 3.9 Aging genes can induce cell apoptosis through the caspase‐8 pathway

**FIGURE 9:**
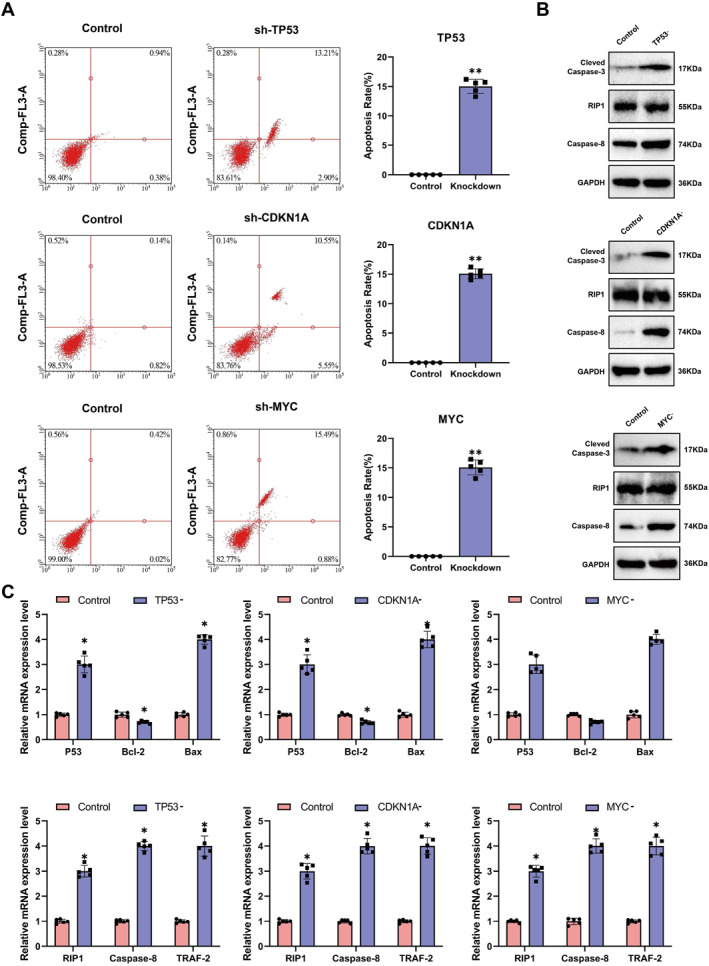
Regulation of cell apoptosis by senescence‐related genes. (A) Impact of gene knockout on cell apoptosis. (B) Influence of gene knockout on key proteins of the Caspase‐8 pathway and cell apoptosis. (C) Effects of gene knockout on mRNA levels of key proteins of the Caspase‐8 pathway and cell apoptosis. Significance levels are denoted as follows: *p ≤ 0.05; **p ≤ 0.01; and ns: *p* > 0.05.

Cell‐based assays showed that knockout of Trp53, Cdkn1a, and Myc significantly increased apoptosis compared to the control group (Figure 9A), consistent with in vivo findings. Western blot analysis revealed elevated expression of RIP1, Caspase‐8, and Caspase‐3 proteins in the knockout groups, whereas BID levels showed no notable change (Figure 9B). mRNA expression patterns of apoptosis‐related genes were consistent with protein results (Figure 9C).

**FIGURE 10:**
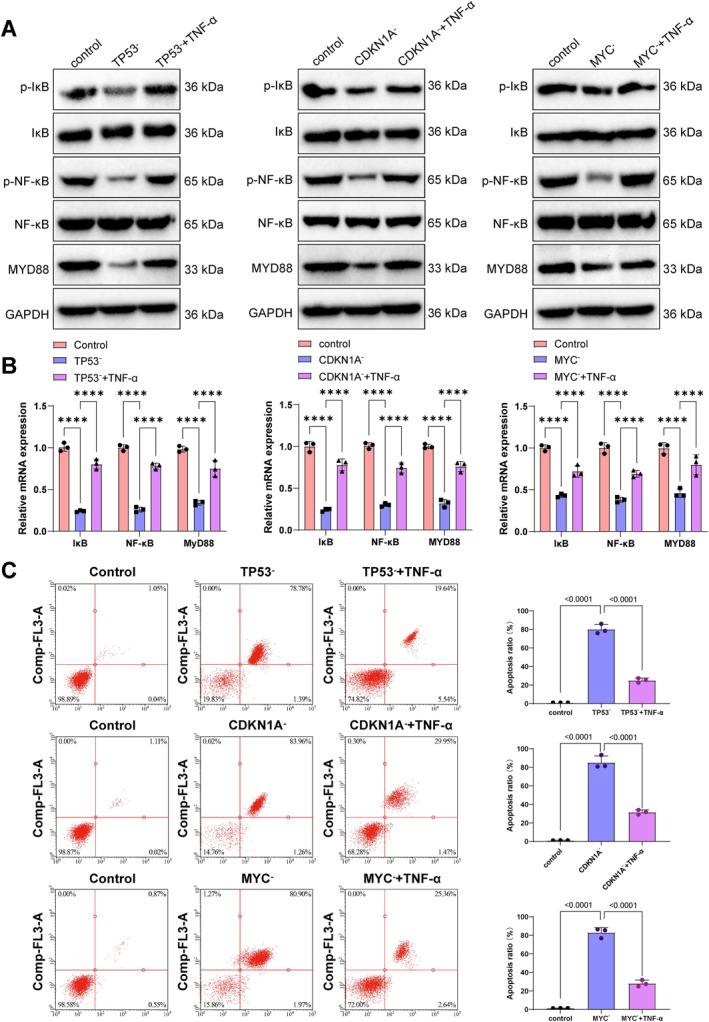
Senescence‐related genes regulate apoptosis via the NF‐κB signaling pathway. (A) Western blot analysis of key proteins in the NF‐κB signaling pathway across different groups. (B) RT‐qPCR analysis of mRNA expression levels of key NF‐κB signaling components in each group. (C) Flow cytometric analysis of apoptosis levels in cells from different groups. Statistical significance is indicated as follows: ****p < 0.0001.

To investigate the underlying mechanism, cells were treated with TNF‐α to activate the NF‐κB signaling pathway. Western blot results showed that expression of NF‐κB pathway proteins was altered following senescence gene knockout and partially restored upon TNF‐α stimulation (Figure 10A), with qPCR results supporting the same trend (Figure 10B). Apoptosis levels were reduced in the knockout groups following NF‐κB activation, indicating a reversal of the pro‐apoptotic effect (Figure 10C).

## 4 DISCUSSION

Cellular senescence is a state of permanent cell cycle arrest induced by various stressors such as DNA damage and oxidative stress.^35^, ^36^, ^37^ Whereas its primary function is to prevent the aberrant proliferation of damaged cells, senescence is also closely associated with the onset of various aging and age‐related diseases.^35^, ^38^, ^39^ Myopia, a common refractive error, is primarily caused by excessive axial elongation or abnormal corneal curvature, resulting in light being focused in front of the retina and leading to blurred distance vision.^40^, ^41^ Given that ocular development is highly dependent on cellular state and tissue homeostasis, cellular senescence may have a profound impact on eye physiology.

This study reveals potential molecular mechanisms linking cellular senescence to myopia development. Expression changes in senescence‐associated genes may influence disease progression by regulating the cell cycle, apoptosis, and immune cell infiltration in ocular tissues.^42^, ^43^, ^44^ Core genes such as Tp53, Cdkn1a, and Myc show abnormal expression patterns that have been experimentally associated with aggravated myopic phenotypes. These results indicate that senescence and immune‐related processes may jointly promote axial elongation and tissue remodeling, offering novel therapeutic targets.

This study focused on the regulatory mechanisms of senescence‐associated genes in myopia, systematically analyzing the roles of Atm, Ezh2, Cdkn1a, Cdkn2a, Myc, Sirt1, and Tp53 in cell cycle regulation, DNA repair, and oxidative stress response. Atm activates Tp53 via phosphorylation, which in turn upregulates Cdkn1a to induce cell cycle arrest and promote DNA repair, playing a key role in oxidative stress responses.^45^, ^46^ Tp53, known as the “guardian of the genome,” regulates cell cycle and senescence through Cdkn1a and Cdkn2a.^47^ Myc promotes cell proliferation and antagonizes Cdkn1a; its dysregulation may disrupt tissue homeostasis. Sirt1 can enhance antioxidant capacity through deacetylation of Tp53 and other targets, potentially offering protective effects in metabolically active ocular tissues. Together, these genes form a key network essential for maintaining ocular development, homeostasis, and stress responses.

mRNA–miRNA and mRNA–transcription factor network analyses showed that these genes coordinate cell function through multi‐level post‐transcriptional mechanisms. Myc activates proliferative miRNA clusters, such as miR‐17‐92, forming a Myc/E2F/miR‐17‐92 positive feedback loop that drives cell cycle progression.^48^, ^49^, ^50^ Myc also regulates various TFs and downstream miRNAs, broadly impacting cell proliferation, metabolism, and apoptosis. miR‐204 negatively regulates Myc, forming a feedback loop.^51^ Additionally, Myc is involved in ceRNA networks, extending its influence in immune and metabolic regulation. These results highlight that myopia development involves complex multi‐pathway and multi‐level regulatory mechanisms, rather than being driven by a single gene.

Immune infiltration analysis revealed significant enrichment of γδ T cells, NKT cells, and neutrophils in myopic samples, suggesting a chronic inflammatory state in local tissues. The release of pro‐inflammatory cytokines may alter the structure and elasticity of the sclera, thereby promoting axial elongation. PPI network analysis further confirmed the hub roles of Tp53, Cdkn1a, and Myc, which mediate immune regulation via miRNAs and TFs, linking senescence to changes in tissue homeostasis. Based on these findings, we propose the “senescence–immunity axis” hypothesis: cellular senescence induces abnormal immune cell infiltration through senescence‐associated secretory phenotype factors, activating inflammation and tissue remodeling, thereby driving myopia progression. Similar mechanisms have been confirmed in other chronic conditions such as rheumatoid arthritis, asthma, and heart failure,^52^, ^53^, ^54^ supporting the generalizability of this model.

Although the same transcriptomic dataset was used,^55^ previous studies focused on immune microenvironment alterations and did not identify core senescence factors such as Tp53 and Cdkn1a. By approaching the problem from a senescence perspective and integrating multi‐component networks, this study identified the upstream regulatory roles of key genes in immune modulation, suggesting their cooperative regulation may be a critical driver of immune imbalance. These findings underscore the value of integrating senescence and immune mechanisms in myopia research and offer a potential basis for targeted interventions.

From a scientific perspective, this study is the first to reveal a molecular link between cellular senescence and myopia, advancing our understanding of its pathophysiology. The identified genes and pathways provide novel targets for future basic research and may reveal previously unknown biomarkers. From a clinical standpoint, these discoveries could foster the development of new therapeutic strategies, especially for treating age‐related visual deterioration, potentially slowing or reversing myopia progression.

Although this study provides new insights into the relationship between cellular senescence and myopia, some limitations remain. First, although bioinformatics analysis has identified key genes related to myopia, the specific mechanisms of these genes still need to be explored further. Additionally, this study primarily relied on mouse models, and the sample size was small; future research should validate these findings in a broader range of animal models and clinical samples. Furthermore, we have only explored gene functions through transcriptomics and PPI network analysis without fully considering the potential effects of epigenetic and post‐transcriptional regulatory mechanisms. Future research should integrate these aspects to further refine our understanding of the molecular mechanisms underlying myopia.

Future research can further investigate the interactions between these senescence‐related genes and environmental factors (such as visual load and eye usage habits) and validate their potential as predictive tools and therapeutic targets in clinical settings. Combining broader genetic backgrounds and environmental variables will help deepen our understanding of the pathological mechanisms of myopia, ultimately advancing the development of novel therapeutic strategies.

## 5 CONCLUSION

This study used an integrative genomics approach to explore the role of cellular aging in myopia. Transcriptomic analysis revealed senescence‐related gene dysregulation in myopia versus controls. PPI networks identified Tp53/Cdkn1a/Myc as central regulators, with mRNA–miRNA–TF networks elucidating their post‐transcriptional control. Immune profiling showed altered γδ T cell and neutrophil infiltration, suggesting immune–senescence crosstalk. Future studies should combine murine models and multi‐omics to dissect how immune–senescence interactions drive myopia progression.

## AUTHOR CONTRIBUTIONS

**Wangming Su**: Conceptualization; data curation; formal analysis; writing—original draft. **Pinsheng Qiu**: Methodology; validation; software; visualization. **Yanling Li**: Investigation; resources; data interpretation. **Ping Xie**: Experimental validation; animal studies; data acquisition. **Xiaoyong Yuan**: Conceptualization; supervision; funding acquisition; writing—review and editing; project administration.

## CONFLICT OF INTEREST STATEMENT

The authors declare no conflicts of interest.

## ETHICS STATEMENT

All animal experiments were approved by the Animal Ethics Committee of the Second Hospital of Longyan City (LYEYEC2023‐039).

## Supporting information

Supporting Information S1

## Data Availability

All data can be provided as needed.
