# Supplementary material for: Integrated genomics reveals cellular senescence‐driven molecular networks and immune crosstalk in myopia pathogenesis
Source: J Cell Commun Signal. 2025 Sep 21;19(3):e70045. doi: 10.1002/ccs3.70045 (PMC12450603; doi:10.1002/ccs3.70045)
Supplement: Supplementary file 1 — Supporting Information S1 [file CCS3-19-e70045-s001.docx]

**
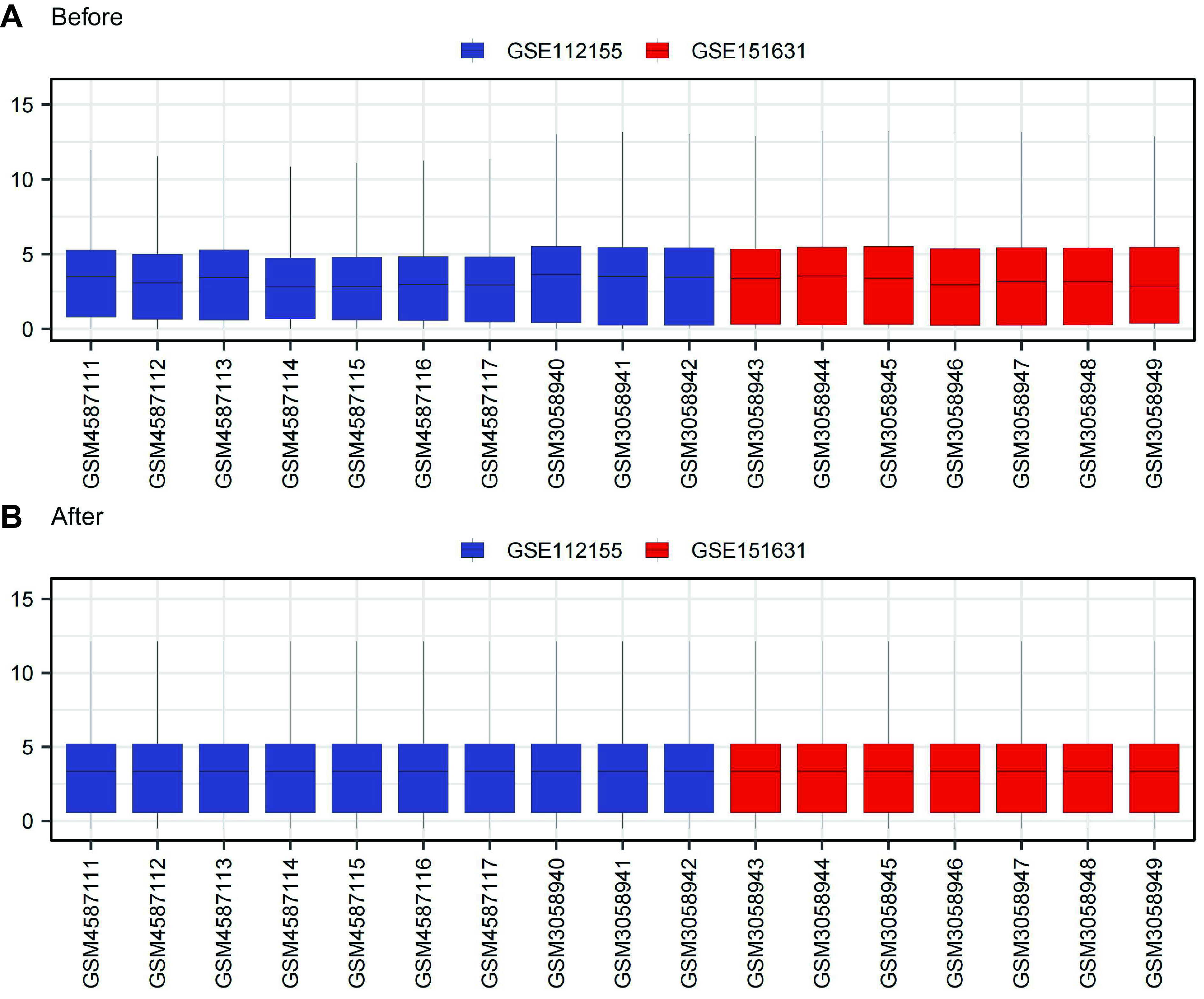
**

**Figure S1. Schematic Diagram of the Merged Dataset.**

Note: (A) Schematic diagram of the merged dataset before data correction. (B) Schematic diagram of the merged dataset after data correction.

**
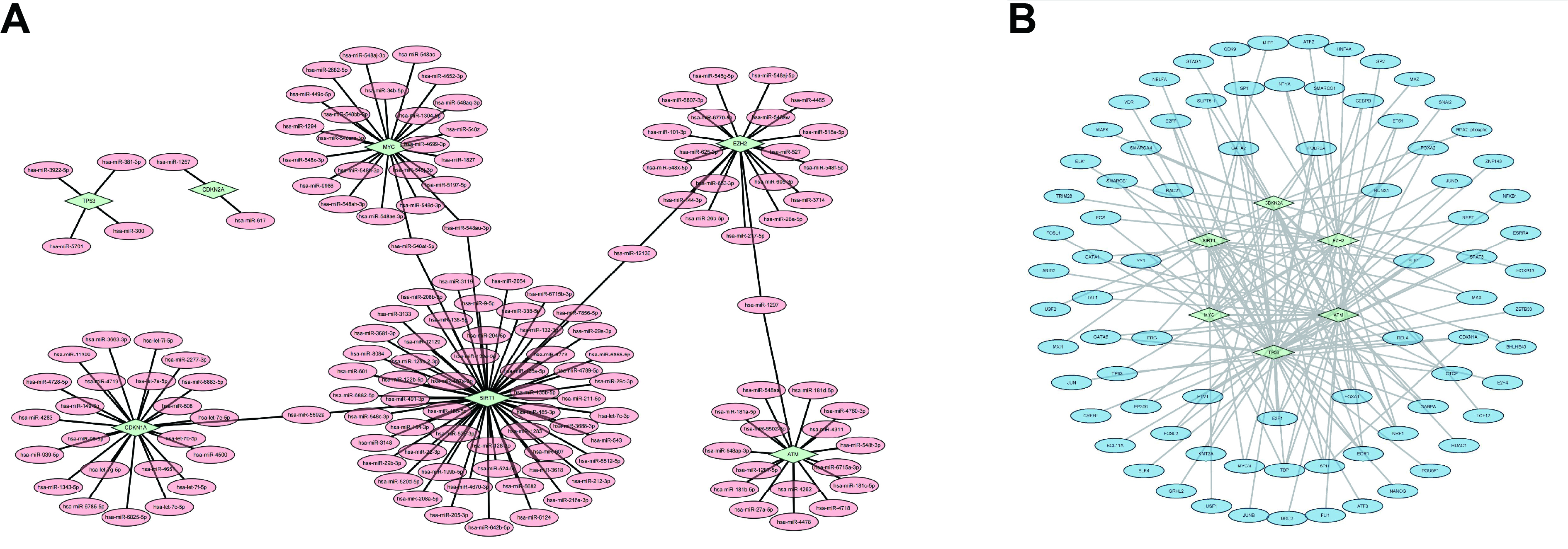
**

**Figure S2. mRNA-miRNA and mRNA-TF Interaction Networks of Central Genes.**

Note: (A) mRNA-miRNA interaction network of central genes. (B) mRNA-TF interaction network of central genes. In the mRNA-miRNA interaction network (A), green diamonds represent mRNA; pink circles represent miRNA. In the mRNA-TF interaction network (B), green diamonds represent mRNA; blue circles represent TFs.

**
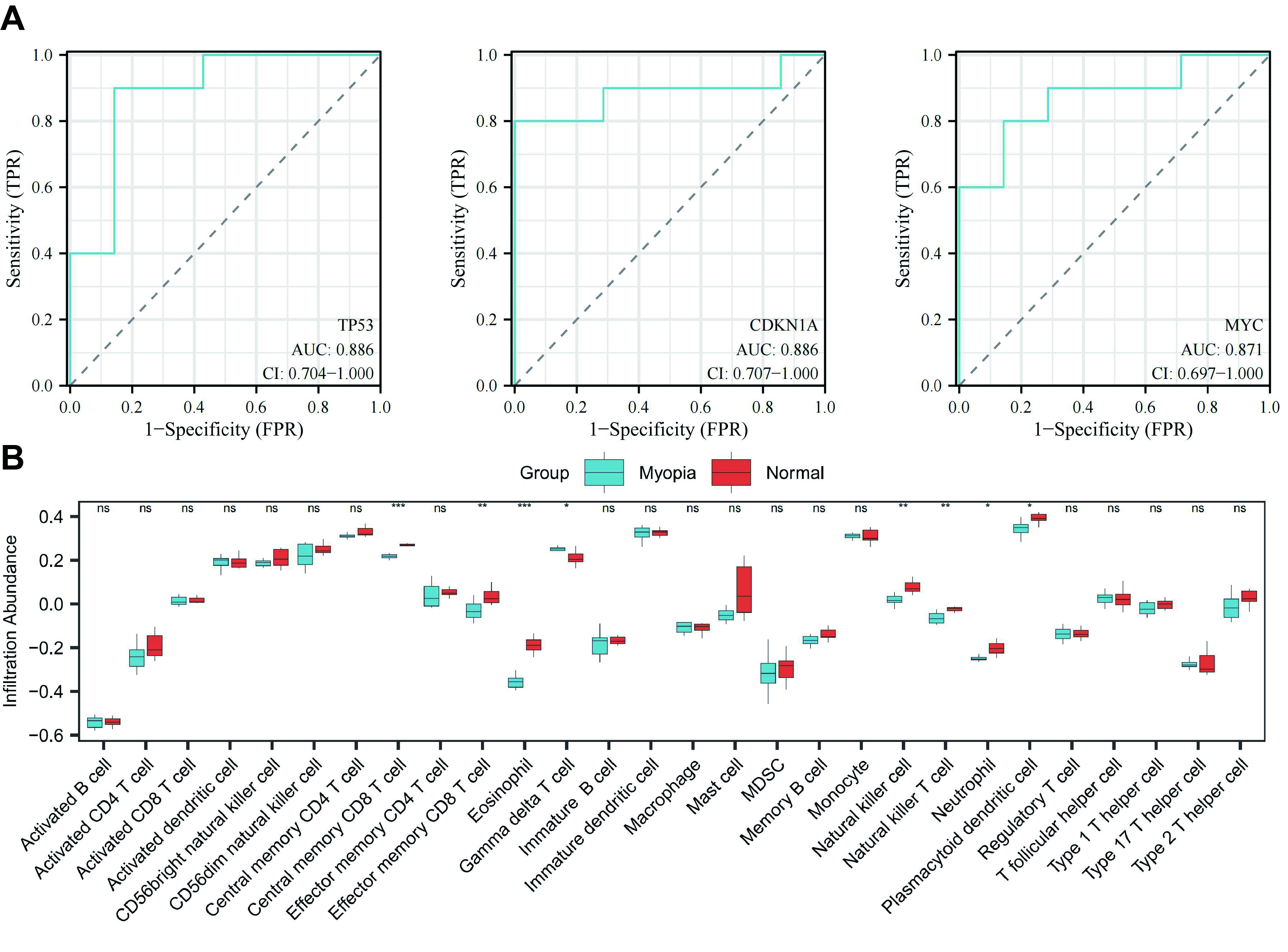
**

**Figure S3. Group Comparison of Diagnostic Performance of Central Genes and Immune Cell Infiltration in Myopia and Normal Vision Groups.**

Note: (A) ROC curve results for 10 central genes (CPT1C, EIF4E, IL1B, IRS1, MAVS, PDK4, PFKFB2, PFKP, SOD2, UCP3) with normal and myopia as outcome variables. Significance levels are denoted as follows: *: p ≤0.05, **: p ≤0.01. (B) Comparative plot of ssGSEA immune cell infiltration analysis results in the dataset samples.

**Table S1. Network Topological Scores of Candidate CSRDEGs Based on MCC, MNC, Degree, EPC, and Closeness Algorithms in CytoHubba**

| **Node name** | **MCC** | **MNC** | **Degree** | **EPC** | **Closeness** |
| --- | --- | --- | --- | --- | --- |
| WRN | 10128 | 10 | 10 | 7.416 | 18.5 |
| TP53 | 2.406762029E9 | 24 | 25 | 10.765 | 26 |
| TERF2 | 50 | 6 | 6 | 5.083 | 16.33333 |
| SP1 | 7660800 | 13 | 13 | 8.643 | 19.83333 |
| SIRT6 | 85680 | 10 | 10 | 7.872 | 18.5 |
| SIRT1 | 2.40676128E9 | 21 | 21 | 10.6 | 24 |
| RSL1D1 | 1 | 1 | 1 | 1.988 | 13.66667 |
| RB1 | 1.44829944E9 | 18 | 18 | 10.087 | 22.5 |
| POT1 | 24 | 4 | 4 | 3.529 | 14.66667 |
| PML | 40322 | 9 | 9 | 6.749 | 18 |
| NUAK1 | 2 | 2 | 2 | 2.811 | 14.33333 |
| MYC | 2.406762E9 | 22 | 22 | 10.563 | 24.5 |
| MAPK14 | 4359600 | 13 | 13 | 8.796 | 20 |
| IL1A | 10080 | 8 | 8 | 6.466 | 17.33333 |
| ID1 | 720 | 6 | 6 | 5.501 | 16.33333 |
| FOS | 4.866732E8 | 17 | 17 | 9.535 | 22 |
| EZH2 | 2.4023916E9 | 19 | 19 | 10.173 | 23 |
| E2F1 | 2.40226632E9 | 17 | 17 | 9.624 | 22 |
| CDKN2B | 1.9196352E9 | 15 | 15 | 9.256 | 21 |
| CDKN2A | 2.406762001E9 | 22 | 23 | 10.689 | 25 |
| CDKN1B | 1.4410368E9 | 16 | 16 | 9.283 | 21.5 |
| CDKN1A | 2.4067116E9 | 20 | 20 | 10.63 | 23.5 |
| CDK6 | 2.395008E9 | 15 | 15 | 9.58 | 21 |
| CDK4 | 2.40662016E9 | 17 | 17 | 9.758 | 22 |
| BMI1 | 9.5836608E8 | 14 | 14 | 9.255 | 20.5 |
| ATM | 2.40909045E9 | 21 | 21 | 10.362 | 24 |

Note: WRN (Werner syndrome RecQ like helicase), TP53 (Tumor protein p53), TERF2 (Telomeric repeat binding factor 2), SP1 (Sp1 transcription factor), SIRT6 (Sirtuin 6), SIRT1 (Sirtuin 1), RSL1D1 (Ribosomal L1 domain containing 1), RB1 (RB transcriptional corepressor 1), POT1 (Protection of telomeres 1), PML (Promyelocytic leukemia protein), NUAK1 (NUAK family kinase 1), MYC (MYC proto-oncogene, bHLH transcription factor), MAPK14 (Mitogen-activated protein kinase 14), IL1A (Interleukin 1 alpha), ID1 (Inhibitor of DNA binding 1), FOS (Fos proto-oncogene, AP-1 transcription factor subunit), EZH2 (Enhancer of zeste 2 polycomb repressive complex 2 subunit), E2F1 (E2F transcription factor 1), CDKN2B (Cyclin dependent kinase inhibitor 2B), CDKN2A (Cyclin dependent kinase inhibitor 2A), CDKN1B (Cyclin dependent kinase inhibitor 1B), CDKN1A (Cyclin dependent kinase inhibitor 1A), CDK6 (Cyclin dependent kinase 6), CDK4 (Cyclin dependent kinase 4), BMI1 (BMI1 proto-oncogene, polycomb ring finger), and ATM (ATM serine/threonine kinase).
